# Inflammatory biomarkers in Alzheimer's disease plasma

**DOI:** 10.1016/j.jalz.2019.03.007

**Published:** 2019-06

**Authors:** Angharad R. Morgan, Samuel Touchard, Claire Leckey, Caroline O'Hagan, Alejo J. Nevado-Holgado, NIMA Consortium, Frederik Barkhof, Lars Bertram, Olivier Blin, Isabelle Bos, Valerija Dobricic, Sebastiaan Engelborghs, Giovanni Frisoni, Lutz Frölich, Silvey Gabel, Peter Johannsen, Petronella Kettunen, Iwona Kłoszewska, Cristina Legido-Quigley, Alberto Lleó, Pablo Martinez-Lage, Patrizia Mecocci, Karen Meersmans, José Luis Molinuevo, Gwendoline Peyratout, Julius Popp, Jill Richardson, Isabel Sala, Philip Scheltens, Johannes Streffer, Hikka Soininen, Mikel Tainta-Cuezva, Charlotte Teunissen, Magda Tsolaki, Rik Vandenberghe, Pieter Jelle Visser, Stephanie Vos, Lars-Olof Wahlund, Anders Wallin, Sarah Westwood, Henrik Zetterberg, Simon Lovestone, B. Paul Morgan, Annex: NIMA–Wellcome Trust Consortium for Neuroimmunology of Mood Disorders and Alzheimer's Disease, Edward T. Bullmore, Junaid Bhatti, Samuel J. Chamberlain, Marta M. Correia, Anna L. Crofts, Amber Dickinson, Andrew C. Foster, Manfred G. Kitzbichler, Clare Knight, Mary-Ellen Lynall, Christina Maurice, Ciara O'Donnell, Linda J. Pointon, Peter St George Hyslop, Lorinda Turner, Petra Vertes, Barry Widmer, Guy B. Williams, B. Paul Morgan, Claire A. Leckey, Angharad R. Morgan, Caroline O'Hagan, Samuel Touchard, Jonathan Cavanagh, Catherine Deith, Scott Farmer, John McClean, Alison McColl, Andrew McPherson, Paul Scouller, Murray Sutherland, H.W.G.M. Boddeke, Jill C. Richardson, Shahid Khan, Phil Murphy, Christine A. Parker, Jai Patel, Declan Jones, Peter de Boer, John Kemp, Wayne C. Drevets, Jeffrey S. Nye, Gayle Wittenberg, John Isaac, Anindya Bhattacharya, Nick Carruthers, Hartmuth Kolb, Carmine M. Pariante, Federico Turkheimer, Gareth J. Barker, Heidi Byrom, Diana Cash, Annamaria Cattaneo, Antony Gee, Caitlin Hastings, Nicole Mariani, Anna McLaughlin, Valeria Mondelli, Maria Nettis, Naghmeh Nikkheslat, Karen Randall, Hannah Sheridan, Camilla Simmons, Nisha Singh, Victoria Van Loo, Marta Vicente-Rodriguez, Tobias C. Wood, Courtney Worrell, Zuzanna Zajkowska, Niels Plath, Jan Egebjerg, Hans Eriksson, Francois Gastambide, Karen Husted Adams, Ross Jeggo, Christian Thomsen, Jan Torleif Pederson, Brian Campbell, Thomas Möller, Bob Nelson, Stevin Zorn, Jason O'Connor, Mary Jane Attenburrow, Alison Baird, Jithen Benjamin, Stuart Clare, Philip Cowen, I-Shu (Dante) Huang, Samuel Hurley, Helen Jones, Simon Lovestone, Francisca Mada, Alejo Nevado-Holgado, Akintayo Oladejo, Elena Ribe, Katy Smith, Anviti Vyas, Zoe Hughes, Rita Balice-Gordon, James Duerr, Justin R. Piro, Jonathan Sporn, V. Hugh Perry, Madeleine Cleal, Gemma Fryatt, Diego Gomez-Nicola, Renzo Mancuso, Richard Reynolds, Neil A. Harrison, Mara Cercignani, Charlotte L. Clarke, Elizabeth Hoskins, Charmaine Kohn, Rosemary Murray, Lauren Wilcock, Dominika Wlazly, Howard Mount

**Affiliations:** a Systems Immunity Research Institute and UK Dementia Research Institute Cardiff, School of Medicine, Cardiff University, Cardiff, UK; b Department of Psychiatry, University of Oxford, Oxford, UK; c Department of Radiology and Nuclear Medicine, VU University Medical, Amsterdam, the Netherlands; d UCL Institutes of Neurology and Healthcare Engineering, University College London, London, UK; e Max Planck Institute for Molecular Genetics, Berlin, Germany; f Aix-Marseille University, APHM, Institute Neurosci System, Pharmacology, Marseille, France; g Alzheimer Centrum Limburg, Maastricht University, Maastricht, the Netherlands; h Lübeck Interdisciplinary Platform for Genome Analytics, University of Lübeck, Lübeck, Germany; i Department of Neurology, Hospital Network Antwerp (ZNA), Antwerp, Belgium; j Reference Center for Biological Markers of Dementia, Institute Born-Bunge, Antwerp, Belgium; k University of Geneva, Geneva, Switzerland; l IRCCS Istituto Centro San Giovanni di Dio Fatebenefratelli, Brescia, Italy; m Department of Geriatric Psychiatry, Zentralinstitut für Seelische Gesundheit, University of Heidelberg, Mannheim, Germany; n Department of Neurosciences, Laboratory for Cognitive Neurology, KU Leuven, Leuven, Belgium; o Division of Clinical Geriatrics, Department of Neurobiology, Caring Sciences and Society, Karolinska Institutet, Stockholm, Sweden; p University of Gothenburg, Institute of Neuroscience and Physiology, Gothenburg, Sweden; q Department of Old Age Psychiatry & Psychotic Disorders, Medical University of Lodz, Lodz, Poland; r School of Public Health, Imperial College London, London, UK; s Department of Neurology, Hospital de la Santa Creu i Sant Pau, Barcelona, Spain; t CITA-Alzheimer Foundation, San Sebastian, Spain; u Department of Medicine, Institute of Gerontology and Geriatrics, University of Perugia, Perugia, Italy; v Barcelona Beta Brain Research Center, Unversitat Pompeu Fabra, Barcelona, Spain; w Department of Psychiatry, Old Age Psychiatry, Lausanne University Hospital, Lausanne, Switzerland; x Hopitaux Universitaires Geneve and Universite de Geneve, Geneva, Switzerland; y Neurosciences Therapeutic Area, GlaxoSmithKline R&D, Stevenage, UK; z Memory Unit, Neurology Department, Hospital de la Santa Creu i Sant Pau, Barcelona, Spain; aa Alzheimer Center, Amsterdam University Medical Centers, Vrije Universiteit, Amsterdam, the Netherlands; bb Reference Center for Biological Markers of Dementia (BIODEM), Institute Born-Bunge, University of Antwerp, Antwerp, Belgium; cc Institute of Clinical Medicine, Neurology, University of Eastern Finland, Kuopio, Finland; dd Center for Research and Advanced Therapies. CITA-Alzheimer Foundation, San Sebastian, Spain; ee University Hospital Leuven, Leuven, Belgium; ff 1st Department of Neurology, AHEPA University Hospital, Makedonia, Thessaloniki, Greece; gg Department of Clinical Chemistry, Neurochemistry lab, Amsterdam University Medical Centers, Amsterdam, the Netherlands; hh Department of Psychiatry & Neuropsychology, School for Mental Health and Neuroscience, Maastricht University, Maastricht, the Netherlands; ii NVS-Department, Section of Clinical Geriatrics, Karolinska Institutet, Huddinge, Sweden; jj Section for Psychiatry and Neurochemistry, Institute of Neuroscience and Physiology, University of Gothenburg Sahlgrenska Academy, Gothenburg, Sweden; kk Clinical Neurochemistry Lab, Institute of Neuroscience and Physiology, Sahlgrenska University Hospital, Mölndal, Sweden; ll Institute of Neuroscience and Physiology, Department of Psychiatry and Neurochemistry, University of Gothenburg, Mölndal, Sweden; mm Department of Molecular Neuroscience, UCL Institute of Neurology, London, UK; nn UK Dementia Research Institute, London, UK

**Keywords:** Alzheimer's disease, Biomarker, Plasma, Inflammation, Complement

## Abstract

**Introduction:**

Plasma biomarkers for Alzheimer's disease (AD) diagnosis/stratification are a “Holy Grail” of AD research and intensively sought; however, there are no well-established plasma markers.

**Methods:**

A hypothesis-led plasma biomarker search was conducted in the context of international multicenter studies. The discovery phase measured 53 inflammatory proteins in elderly control (CTL; 259), mild cognitive impairment (MCI; 199), and AD (262) subjects from AddNeuroMed.

**Results:**

Ten analytes showed significant intergroup differences. Logistic regression identified five (FB, FH, sCR1, MCP-1, eotaxin-1) that, age/APOε4 adjusted, optimally differentiated AD and CTL (AUC: 0.79), and three (sCR1, MCP-1, eotaxin-1) that optimally differentiated AD and MCI (AUC: 0.74). These models replicated in an independent cohort (EMIF; AUC 0.81 and 0.67). Two analytes (FB, FH) plus age predicted MCI progression to AD (AUC: 0.71).

**Discussion:**

Plasma markers of inflammation and complement dysregulation support diagnosis and outcome prediction in AD and MCI. Further replication is needed before clinical translation.

## 1 Introduction

Alzheimer's disease (AD) is a complex neurodegenerative disorder that develops gradually and progressively, with symptoms progressing over time from mild forgetfulness to severe mental impairment. Early diagnosis is an essential requirement for effective intervention but is challenging because of current reliance on clinical observation and cognitive testing, with diagnosis confirmed postmortem by demonstrating typical AD brain pathology. Biomarkers of early disease might address this challenge and are thus an urgent unmet need.

Currently, cerebrospinal fluid (CSF) levels of amyloid β (Aβ) fragments and hyperphosphorylated or total tau are the most widely used biomarkers for AD [1], [2]; however, diagnostic accuracy varies between centers [3]. Furthermore, lumbar puncture is invasive and difficult to implement in the presymptomatic elderly population. The accessibility and practicability of obtaining peripheral blood to measure disease biomarkers make this an attractive option for early diagnosis and large-scale screening. Numerous discovery studies for blood-based biomarkers of AD have been reported, but validation and replication remain key challenges and none has yet achieved clinical usefulness [4], [5], [6], [7]. Promising candidates do exist, for example, plasma Aβ42/40 ratio and neurofilament light chain [8], but more work is needed.

Considerable evidence implicates inflammation and complement dysregulation in AD pathogenesis. Genome-wide association studies demonstrated strong associations between AD and common SNPs in the gene encoding the complement regulator clusterin (*CLU*) [9]. A second genome-wide association study replicated the *CLU* association and identified association with an SNP in the *CR1* gene, encoding complement receptor 1 (CR1) [10]. These findings have been robustly replicated in diverse cohorts. Furthermore, pathway analysis has highlighted immunity, inflammation, and complement as key pathways in AD [11], [12], [13]. Other evidence implicating inflammation and complement includes longitudinal studies demonstrating that inflammation occurs years before AD onset [14], [15], and cross-sectional studies reporting increased inflammatory markers in early AD [16]. Plasma markers of inflammation and complement dysregulation may therefore be useful biomarkers of early AD. Indeed, complement proteins, regulators, and activation products were altered in AD plasma and/or CSF [17], and in a systematic review of 21 discovery or panel-based blood proteomic studies, complement was the top implicated pathway across the studies [18].

The underpinning hypothesis of this study is that plasma levels of complement proteins and other inflammatory biomarkers differ between neurologically normal elderly controls (CTL) and those with mild cognitive impairment (MCI) and/or AD, between subjects with MCI and those with AD, and between subjects with MCI destined to rapidly progress to AD (progressors) and those who will not progress (nonprogressors). If proven, then the most informative of these plasma biomarkers can be used to diagnose, stratify, predict disease progression, and/or demonstrate response to intervention in MCI and AD. Analytes were selected based on biological evidence and published studies of inflammatory/complement biomarkers in neurodegeneration. In the discovery phase, we used singleplex and multiplex solid-phase enzyme immunoassays to measure 53 proteins comprising complement components, activation products and regulators, cytokines and chemokines in a large cohort comprising AD, MCI, and CTL samples. Proteins demonstrating association with AD and/or MCI in this discovery sample set were investigated further in two independent replication cohorts.

## 2 Methods

### 2.1 Study population

Discovery phase samples were from AddNeuroMed, a cross-European cohort for biomarker discovery, detailed elsewhere [19], [20]. Informed consent was obtained according to the Declaration of Helsinki (1991), and protocols and procedures were approved by Institutional Review Boards at each collection site. We used 720 plasma samples from the cohort: 262 AD, 199 MCI, and 259 CTL, selected based solely on availability of plasma samples. The replication cohorts comprised (1) 867 plasma samples (88 AD, 425 MCI, 347 CTL) from European Medical Information Framework for Alzheimer's Disease Multimodal Biomarker Discovery (EMIF-AD MBD), a cross-European biomarker discovery cohort [21]; (2) 427 plasma samples (105 AD, 69 MCI, 253 CTL) from Maudsley Biomedical Research Centre Dementia Case Registry (DCR) [22]. In both cases, samples were selected based solely on availability of plasma; plasma was not collected from all individuals in the cohorts and stocks had been exhausted for others. Diagnostic categories were created using similar algorithms in the discovery and replication cohorts [19], [20], [21], [22]. In all cohorts, the definition for CTL was a normal performance on neuropsychological assessment (within 1.5 SD of the average for age, gender, and education). Diagnosis of MCI was made according to the criteria of Petersen [23], and AD-type dementia was diagnosed using the National Institute of Neurological and Communicative Disorders and Stroke–Alzheimer's Disease and Related Disorders Association criteria [24].

Patient data available differed between the cohorts; therefore, a minimal clinical data set was collected and harmonized as described [21]; this data set comprised 1) demographics: age, gender, education; 2) clinical information: diagnosis, medication use, comorbidities, family history of dementia, functional impairment rating; 3) cognitive data: Mini–Mental State Examination, neuropsychological testing. Imaging data and CSF samples were not available for a majority of cases included in the cohorts and so could not be included in the analyses; however, this was not considered an issue given that the aim of the work was to identify plasma markers that correlated with clinical disease status.

### 2.2 Discovery phase assays

In the discovery phase, 53 plasma analytes were measured using commercial and in-house singleplex and multiplex assays on all available samples in duplicate from AddNeuroMed. Plasma clusterin, soluble complement receptor 2, C-reactive protein (CRP), colony-stimulating factor 1 (CSF1), and interleukin-23 (IL-23) were determined using commercially available enzyme-linked immunosorbent assays (clusterin, CRP, CSF1, and IL-23 from R&D systems (Abingdon, UK; cat# DY5874, DY1707, DY216, and DY5265 B) and soluble complement receptor 2 from Sino Biological (Beijing, China; cat# SEKA10811); protocols were as recommended by the manufacturers. Plasma soluble complement receptor 1 (sCR1), C1-inhibitor (C1inh), C5, C9, C1q, factor H-related protein 4 (FHR4), factor H (FH) Y402, and H402 variants were determined using optimized antibody pairs in in-house enzyme-linked immunosorbent assays as described [25]. Ten complement biomarkers were measured using customized V-plex electrochemiluminescence (ECL) immunoassays (MSD; Rockville, Maryland); antibody pairs were developed and optimized in-house. Multiplex 1 comprised abundant analytes C3, C4, factor B (FB), FH, and factor I (FI). Multiplex 2 comprised low-concentration analytes factor D (FD); the activation fragments Bb, C3a, and iC3b; and the terminal complement complex (TCC). A calibration curve comprising five-fold dilutions of a mixture of protein standards was run in duplicate on each plate. ECL signal was measured on the MESO QuickPlex SQ 120 reader (MSD). Data acquisition and analysis was performed using MSD software Discovery workbench 4.0.

The V-Plex Human Cytokine 30-Plex Kit (MSD; cat# K15054D) was used to measure cytokines/chemokines. The kit comprises three 10-plex panels: V-plex Proinflammatory Panel 1 measures interferon γ, interleukin (IL)-1β, IL-2, IL-4, IL-6, IL-8, IL-10, IL-12p70, IL-13, and tumor necrosis factor (TNF)-α in samples diluted 1:2 in proprietary buffer; V-plex cytokine panel 1 measures granulocyte-macrophage colony-stimulating factor, IL-1α, IL-5, IL-7, IL-12/IL-23p40, IL-15, IL-16, IL-17A, TNF-β, and vascular endothelial growth factor–A in samples diluted 1:4; V-plex chemokine panel 1 measures eotaxin-1, macrophage inflammatory protein (MIP)-1β, eotaxin-3, thymus- and activation-regulated chemokine (TARC; CCL17), interferon-γ-inducible protein (IP)-10, MIP-1α, IL-8, MCP-1, macrophage-derived chemokine, and MCP-4 in samples diluted 1:4. All assays were performed according to manufacturer's instructions using ECL detection as mentioned previously. Intra-assay and interassay limits for coefficients of variation (CV) were set at 25%, and data for samples with a CV above this were not included in the analysis.

### 2.3 Replication phase assays

The analytes selected from the discovery phase for replication were sCR1, FB, FH, MCP-1, and eotaxin-1; FI, TCC, clusterin, and C4 were also included to replicate previously reported association of these biomarkers with MCI progression [26], not tested in AddNeuroMed cohort.

MSD U-PLEX custom multiplexing was used in replication phase to build bespoke panels. Plasma levels of low abundance analytes sCR1, MCP-1, eotaxin-1, and TCC were measured in one panel with samples diluted 1:2; plasma levels of high abundance analytes FB, FH, FI, clusterin, and C4 were measured in a second panel with samples diluted 1:2000. Assays were performed according to the manufacturer's instructions using ECL detection. Both panels were run on all EMIF-AD MBD and DCR samples in duplicate. Intra-assay and interassay limits for CV were set at 25% as above.

### 2.4 Statistical analysis

All statistical tests and analyses were performed with R software, including ggplot2, caret, and pROC packages. In all cases, *P* < .05 was considered statistically significant.

#### 2.4.1 Individual analytes

Protein concentrations were determined automatically from standard curves plotted using GraphPad Prism5. Values were adjusted for recruitment center and plasma storage time as described [27] using a generalized linear regression model. All subsequent analyses were performed on generalized linear regression model–adjusted data and log-transformed to achieve normal distribution. In the discovery phase, association of individual analytes with disease status was tested using the Kruskal-Wallis test. Pairwise comparisons were then performed using the Dunn test with Bonferroni correction. For 12 analytes (eotaxin-3, granulocyte-macrophage colony-stimulating factor, IL-1β, IL-2, IL-4, IL-5, IL-7, IL-10, IL12p70, IL-13, MIP-1a, TNFβ), many samples were below assay detection limits; these were analyzed as binary variables (positive or negative) and tested for association with disease status by chi-square test.

#### 2.4.2 Identification of optimal analyte sets

Stepwise logistic regression (SLR) was used to find the analyte set that optimally distinguished between diagnostic groups: CTL versus AD, CTL versus MCI, MCI versus AD. Demographic covariates age, gender, and apolipoprotein E (*APOE*) genotype were controlled for and included in models as potential predictors. For each comparison, the data set was randomly split into training (80%) and validation (20%) sets. The training set was used to select variables and fit the model which was then tested on the validation set using receiver operating curve (ROC) analysis. The models developed for AD versus CTL and MCI versus AD were tested in the replication cohorts using ROC analysis.

#### 2.4.3 Markers of disease progression

Data on MCI progression to AD were only available in a subset of the EMIF-AD MBD; in this case, SLR was used to find the analyte set that best distinguished individuals who subsequently progressed from MCI to AD from nonprogressors. Because the MCI conversion group was relatively small, stepwise selection was performed on the complete data set, followed by ROC analysis with leave-one-out cross-validation. To avoid overfitting, 500 replications of stepwise models were performed on random data subsets, each comprising a training set (80%) for selection and a validation set (20%) for model testing, and ROC analysis performed for each replication. The variables most often selected and significant were identified.

## 3 Results

### 3.1 Individual analytes differ between discovery set groups

**Fig. 1:**
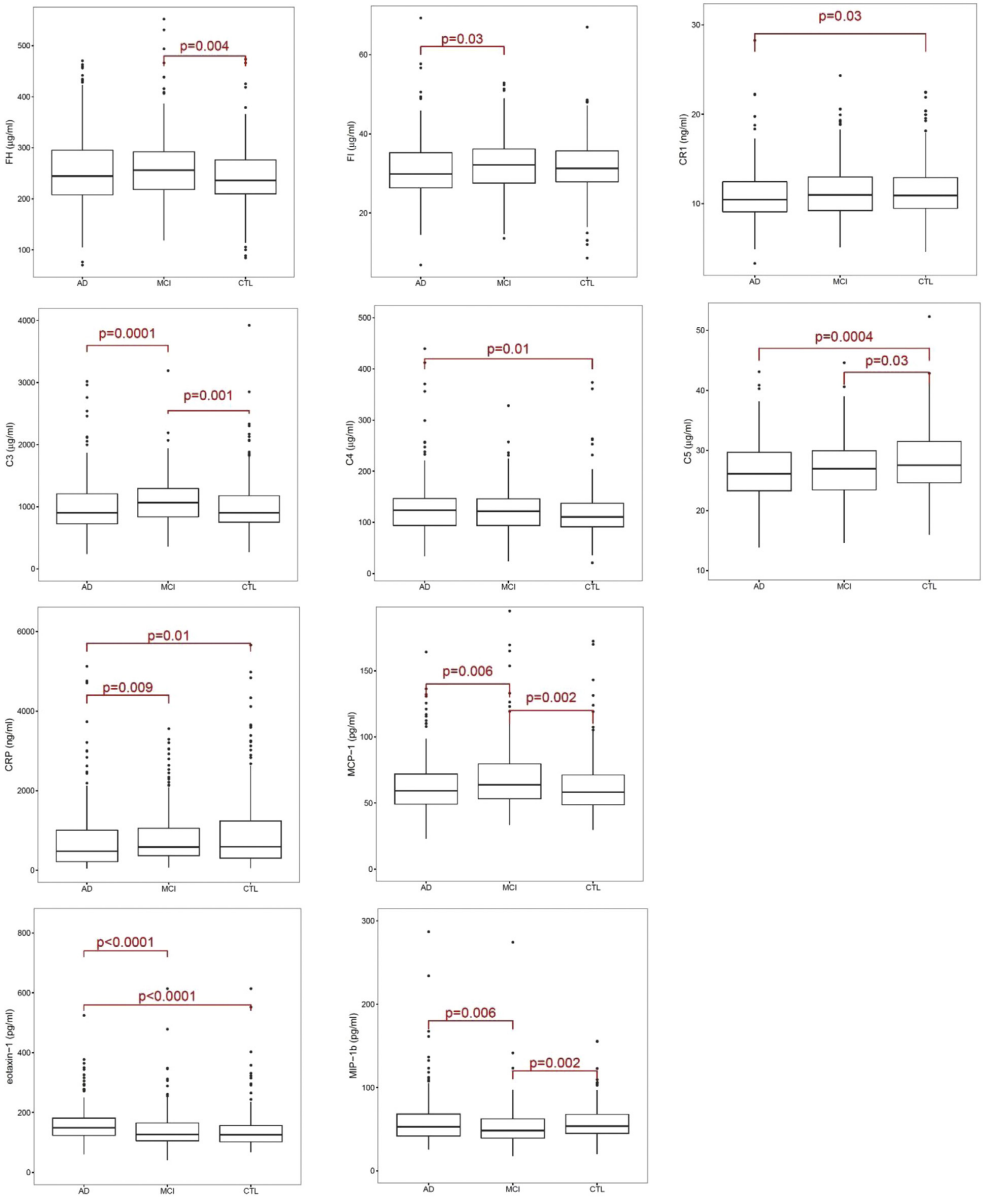
Ten biomarkers associated with diagnosis in the discovery phase. Boxplots for the 10 biomarkers which demonstrated significant differences in concentrations between diagnostic groups (Kruskal-Wallis). The *P* values shown are from the Dunn test with Bonferroni correction for pairwise comparisons; bars indicate significant differences. For graphical convenience and better visualization, high outliers were removed from the boxplots, although all are included in the Kruskal-Wallis analysis. Abbreviation: CRP, C-reactive protein.

Of the 53 plasma proteins measured in the discovery set, 10 demonstrated significant differences between clinical groups (Table 1). Pairwise comparisons (Dunn test with Bonferroni correction) showed (1) for AD versus CTL, increased C4 and eotaxin-1, decreased sCR1, C5, and CRP; (2) for MCI versus CTL, increased FH, C3, and MCP-1, decreased C5 and MIP-1b; (3) for AD versus MCI, increased eotaxin-1 and MIP-1b, decreased FI, C3, CRP, MCP-1 (Table 1; Fig. 1). Of the 12 MSD cytokine/chemokine panel analytes analyzed categorically, none showed significant differences between clinical groups.

**Table 1:** Ten analytes associated with clinical state in the discovery phase

Analyte	Mean ± SD CTL (n = 259)	Mean ± SD MCI (n = 199)	Mean ± SD AD (n = 262)	*P* value KW test	*P* value AD vs. CTL	*P* value AD vs. MCI	*P* value MCI vs. CTL
FH (μg/ml)	241.5 (56.4)	262.7 (71.8)	258.2 (73.0)	.01	ns	ns	.004
FI (μg/ml)	31.5 (7.0)	32.2 (6.9)	31.0 (7.5)	.049	ns	.03	ns
sCR1 (ng/ml)	11.52 (3.03)	11.43 (3.10)	10.88 (3.01)	.043	.03	ns	ns
C3 (μg/ml)	1042.7 (553.4)	1105.0 (377.4)	1004.2 (435.4)	<.0001	ns	.0001	.001
C4 (μg/ml)	351.6 (129.6)	370.8 (136.2)	386.1 (159.3)	.01	.01	ns	ns
C5 (μg/ml)	84.9 (16.2)	81.0 (14.7)	79.8 (14.7)	.001	.0004	ns	.03
CRP (ng/ml)	996.8 (1145.6)	841.3 (711.1)	761.1 (810.5)	.007	.01	.09	ns
MCP-1 (pg/ml)	63.1 (22.5)	68.5 (24.5)	63.0 (20.4)	.009	ns	.006	.002
Eotaxin-1 (pg/ml)	141.6 (65.0)	143.3 (66.2)	162.5 (78.7)	<.0001	<.0001	<.0001	ns
MIP-1b (pg/ml)	58.9 (29.2)	58.1 (55.2)	63.1 (56.2)	.007	ns	.006	.002

Abbreviations: AD, Alzheimer's disease; CRP, C-reactive protein; CTL, control; KW, Kruskal-Wallis; MCI, mild cognitive impairment; ns, not significant; SD, standard deviation.

NOTE. Ten analytes showed statistically significant differences in concentration between clinical groups in the discovery phase. The table shows means and standard deviations, KW test *P* value, and Dunn test *P* values for each analyte.

### 3.2 Developing models to differentiate groups

#### 3.2.1 AD from CTL

**Fig. 2:**
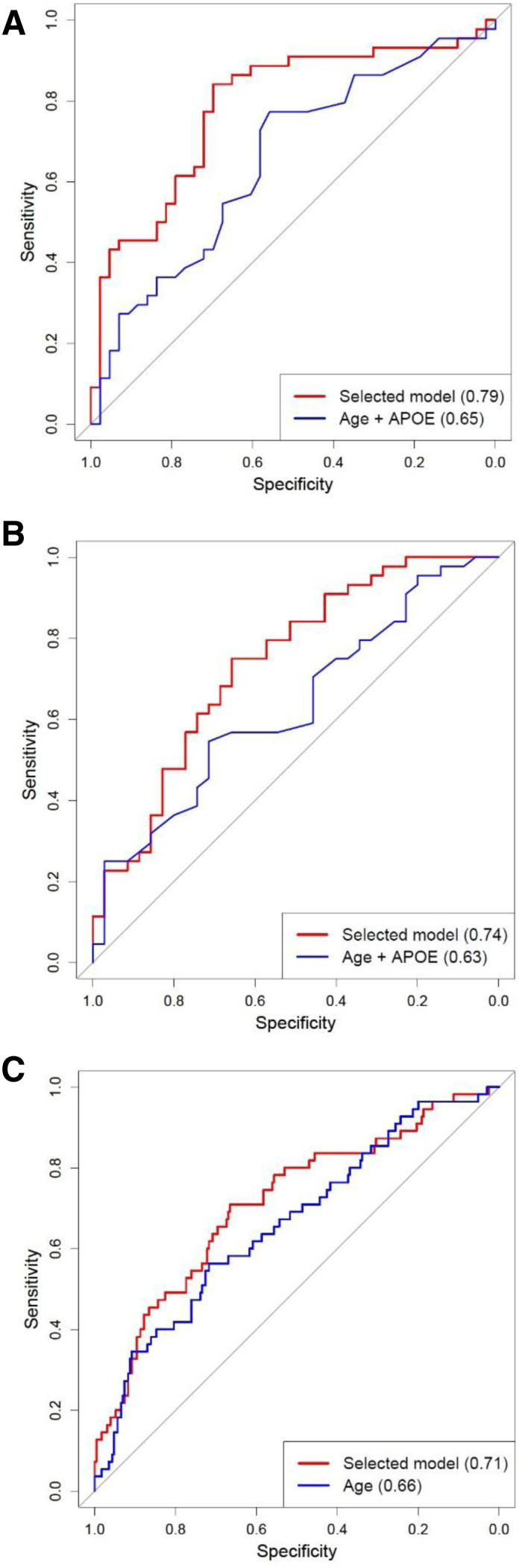
Receiver operating characteristic (ROC) curves for models distinguishing clinical state or predicting progression. ROC curves were generated representing models which best differentiated AD from controls (A) or AD from MCI (B) in the discovery phase and predicted progression or nonprogression in the EMIF cohort (C). In each case, the area under the curve (AUC) for the selected model was calculated, and compared to that for the significant covariables alone, age + APOE ε4 in (A) and (B), age alone in (C). (A) Shows that a model including FB, FH, sCR1, MCP-1, and eotaxin-1, along with the covariables age and APOE genotype, differentiated AD and CTL with a predictive power (AUC) of 0.79 (red line), significantly better than the covariables alone (AUC 0.65; blue line). (B) Shows that a model including sCR1, MCP-1, and eotaxin-1, along with the covariables age and APOE genotype, differentiated AD and MCI with AUC of 0.74 (red line), significantly better than the covariables alone (AUC 0.63; blue line). (C) Shows that a model including FB and FH along with age as covariable differentiated MCI progressors and nonprogressors with AUC of 0.71 (red line). The predictive power was significantly greater than that obtained using the covariable alone (AUC 0.66; blue line). Abbreviations: AD, Alzheimer's disease; APOE, apolipoprotein E; CTL, control; MCI, mild cognitive impairment.

Stepwise selection demonstrated strong interdependence between some analytes and revealed other analytes that significantly and independently contributed to distinguishing clinical groups. SLR modeling was used to identify the most predictive set of analytes. A model combining FB, FH, sCR1, MCP-1, and eotaxin-1 with covariates age and APOε4 status best differentiated AD versus CTL. FH and eotaxin-1 were higher and FB, CR1, and MCP-1 were lower in AD compared to CTL. Diagnostic accuracy in distinguishing CTL from AD was moderate (AUC 0.79); 77% of samples were predicted correctly with 84% sensitivity and 70% specificity (Fig. 2A; Table 2). This model was tested in the replication cohorts. In EMIF-AD MBD, comprising 867 plasma samples (88 AD, 425 MCI, 347 CTL), the model strongly replicated (AD vs. CTL; AUC 0.81), correctly predicting 76% of samples with 73% sensitivity and 77% specificity. In DCR, comprising 427 samples (105 AD, 69 MCI, 253 CTL), the model performed poorly (AD vs. CTL; AUC 0.58).

**Table 2:** Multivariate models for distinguishing between diagnostic groups

Predictor	AD vs. CTL		AD vs. MCI	
	LogOR (95% CI)	*P* value	LogOR (95% CI)	*P* value
Intercept	−13.49 (−27.16; 0.17)	.05	−3.62 (−7.89; 0.65)	.10
Age	0.07 (0.04; 0.12)	.00005	0.06 (0.02; 0.10)	.002
1 APOE ε4	0.74 (0.22; 1.25)	.005	0.41 (−0.10; 0.92)	.12
2 APOE ε4	2.03 (1.0; 3.05)	.0001	1.99 (0.86; 3.13)	.0006
Eotaxin-1	1.56 (0.78; 2.35)	.00009	1.74 (0.97; 2.52)	.00001
MCP-1	−1.31 (−2.21; 0.40)	.0005	−1.91 (−2.82; −1.01)	.00003
sCR1	−0.90 (−1.85; 0.06)	.067	−1.36 (−2.30; −0.41)	.005
FH	2.85 (1.42; 4.27)	.00009	n/a	n/a
FB	−2.33 (−3.60; −1.06)	.0003	n/a	n/a

Abbreviations: AD, Alzheimer's disease; APOE, apolipoprotein E; CTL, control; MCI, mild cognitive impairment; logOR (95% CI), log odds ratio of the predictor and their 95% confidence interval; Intercept, log odds ratio if the predictors are equal to 0; 1 APOE E4/2 APOE E4: log odds ratio of possessing 1 or 2 ε4 alleles compared to possessing no ε4 allele; n/a, predictors not included in the given model.

NOTE. The table summarizes the selected logistic regression models derived from the AddNeuroMed discovery cohort, AD versus CTL in the left panel, AD versus MCI in the right panel.

#### 3.2.2 AD from MCI

A model combining sCR1, MCP-1, and eotaxin-1 with age and APOε4 optimally differentiated AD and MCI (AUC 0.74), correctly predicting 71% of samples with 75% sensitivity and 66% specificity (Fig. 2B; Table 2). FH and eotaxin-1 were higher and FB, sCR1, and MCP-1 were lower in AD compared to MCI samples. The model replicated in EMIF-AD MBD (AUC 0.67), correctly predicting 61% of samples with 71% sensitivity and 59% specificity. In DCR samples, the model performed poorly (AUC 0.56).

#### 3.2.3 MCI from CTL

The optimal model to differentiate MCI from CTL comprised 15 analytes, each providing weak and independent predictive value. Smaller analyte sets were poor predictors (details not shown). We concluded that there was no reliable and practicable biomarker set from the analytes measured that distinguished MCI and CTL.

#### 3.2.4 MCI progressors from nonprogressors

Baseline samples from 285 individuals with MCI who had either progressed to AD when reassessed 12 months later (progressors; 55) or had remained stable over this period (nonprogressors; 230) were compared in EMIF-AD MBD. Of the nine analytes measured, only two, FB (higher in progressors) and FH (lower in progressors), were significantly different between progressors and nonprogressors. A model combining these two analytes with age, the only significant covariable, was moderately predictive (AUC 0.71); 67% of samples correctly predicted, sensitivity 71%, specificity 67% (Table 3). In the 500 replications of stepwise models, age and FH were always selected and significant 499 times, FB was selected 414 times and significant 309 times. No other analyte was selected more than 67 times. The average AUC for the 500 replications was 0.69 (SD 0.09).

**Table 3:** Multivariate model for distinguishing between MCI converters and nonconverters

Predictor	LogOR (95% CI)	*P* value
Intercept	14.13 (−5.77; 34.01)	.16
Age	0.08 (0.04; 0.13)	.00019
FH	−4.15 (−6.24; −2.05)	.00011
FB	2.66 (0.72; 4.60)	.0072

Abbreviations: MCI, mild cognitive impairment; logOR (95% CI), log odds ratio of the predictor and the 95% confidence interval; Intercept, log odds ratio if the predictors are equal to 0.

NOTE. The table summarizes the selected logistic regression model derived from informative samples in the EMIF cohort for MCI converters versus nonconverters.

## 4 Discussion

A plasma biomarker or biomarker set that aids early diagnosis, stratification, prediction of disease course, or monitoring response to therapy in AD is a major unmet need. Numerous studies have sought plasma biomarkers relevant to AD, and many putative plasma protein biomarkers have been proposed (reviewed in the study by Baird et al. [28]); however, none has been robustly replicated. Currently, clinicians rely on neuropsychological testing, a time-consuming tool, to diagnose MCI and AD, with confirmation requiring either expensive neuroimaging (MRI or PET scanning) or invasive lumbar puncture to measure CSF markers of amyloid or tau pathology. These methods are not suitable either for high-volume screening of presymptomatic individuals, required to identify early disease, or frequent monitoring required in assessing response to interventions. Biomarkers informative in CSF are currently difficult to measure in plasma in the routine context [29]. Recent technological advances have improved assay sensitivity, delivering ultrasensitive assays capable of measuring specific amyloid markers in plasma [7], [8], [29], [30], [31]. Promising as these developments are, ultrasensitive assays require expensive purpose-built equipment beyond routine laboratory capacity and currently too costly for large-scale screening.

In this study, we set out to identify plasma analyte sets, measurable using simple multiplex enzyme-linked immunosorbent assay, that differentiated AD, MCI, and CTL groups. We took as a starting point the powerful multisource evidence that inflammation and complement dysregulation were important components of AD pathogenesis [13], [14], [15], [16], [17]. In the discovery phase, we used multiplex and singleplex immunoassays to measure 53 proteins relevant to inflammation and complement dysregulation in a large, well-validated cohort, and identify proteins and/or protein sets associated with AD and/or MCI clinical diagnosis. Ten of the 53 proteins were significantly different between groups of different clinical status; a heterogeneous group of analytes including three complement components (C3, C4, C5), two complement regulators (FH, FI), a soluble form of a complement receptor (sCR1), a classical marker of inflammation (CRP), and three chemokines (eotaxin-1, MCP-1, and MIP-1β). Stepwise selection demonstrated strong interdependence between some analytes, anticipated given that all were selected for relevance to complement and/or inflammation; however, several analytes significantly and independently contributed to distinguishing between clinical groups. To identify the most predictive set, models that tested all combinations of analytes and covariables were generated. The best model for AD versus CTL, including analytes sCR1, FB, FH, eotaxin-1, and MCP-1, with covariables age and *APOE* status, showed an AUC of 0.79 in the discovery cohort, considered “highly predictive” [32]. The best model for AD versus MCI, including analytes sCR1, eotaxin-1, and MCP-1 with covariables age and *APOE* status, yielded an AUC of 0.74, considered “moderately predictive” [32].

Both models were tested in two independent replication cohorts. In the larger of these, EMIF-AD MBD (comprising 867 samples: 88 AD, 425 MCI, 347 CTL), both models replicated, AD versus CTL strongly (AUC 0.81), and AD versus MCI moderately (AUC 0.67). In the smaller DCR cohort (105 AD, 69 MCI, 253 CTL), neither model replicated well (AUC 0.58 for AD vs. CTL; 0.56 for AD vs. MCI). The reasons for failure to replicate in the DCR cohort are unclear; however, this is a relatively small sample set, 60% of which are CTL samples. The strong replication of both models in the larger multicenter EMIF-AD MBD cohort provokes us to suggest that the analytes identified here, perhaps with other promising biomarkers, might provide a basis for a focused, relatively simple and inexpensive plasma multiplex test that could aid diagnosis. Further research in large, well-characterized cohorts to replicate, validate, and extend these findings is needed to deliver a reliable screening tool.

With the exception of FB, each of the analytes selected in the models has previously been associated with AD. sCR1 (reduced in AD vs. CTL and MCI) had not been measured in AD plasma previously but was reported higher in CSF in AD versus CTL [33]. FH (increased in AD vs. CTL) was reported higher in AD plasma in several studies [4], [34], [35], although some reported no difference between clinical groups [36]. Eotaxin-1 (higher in AD plasma vs. CTL and MCI) and MCP-1 (lower in AD plasma vs. CTL and MCI), both C–C chemokine family members, were reported as plasma markers of AD status in several studies [37], [38], [39], [40], [41], [42]; elevated MCP-1 and eotaxin-1 correlated with greater memory impairment in MCI/AD [43].

Several studies have reported plasma biomarkers predictive of MCI progression to AD. An 18-analyte biomarker signature dominated by cytokines/chemokines predicted progression within 5 years with 81% accuracy [44]. A 60-analyte set was predictive of MCI progression to AD with 79% accuracy [45], and a 10-analyte panel, including complement and inflammatory proteins, predicted MCI progression to AD with 87% accuracy [22]. Our published study identified a model comprising three analytes, FI, TCC, and clusterin that, with APOε4 status, predicted progression (AUC 0.86) [26]. To date, none of these findings have been independently replicated. Of the cohorts available to us, only EMIF-AD MBD included data on progression of MCI cases to AD; 19% of informative MCI cases had progressed to AD a year after sampling. Of the 10 analytes measured, two were significantly different; FB levels were higher and FH lower in MCI progressors versus nonprogressors. These two biomarkers together with age (the only significant covariable) predicted MCI conversion with AUC 0.71. Notably, FB is a key enzyme in the complement amplification loop while FH is the critical loop regulator; increased FB and decreased FH seen in progressors would favor amplification, suggesting that amplification loop dysregulation might predispose to progression. We were unable to replicate this finding in other cohorts as data on conversion were not available. Although the model reported for predicting MCI conversion differs from our previous report [26], both identified markers of complement activation/regulation, implying that complement dysregulation is a critical predictor of progression. This finding resonates with preclinical data suggesting that complement and microglial activation play important roles as mediators of neurotoxicity in AD [46]. Further research to replicate and validate markers of complement dysregulation as predictors of progression is required.

There are limitations to the present study. The cohorts were collected across a wide range of centers and without stringent attention to sampling, separation, and storage protocols that are important for complement and other immunity assays; however, despite this suboptimal aspect, characteristic of real-world sample collections, strongly predictive marker sets emerged, increasing the likelihood of utility in clinical practice. For several analytes, the commercial cytokine/chemokine platform was insufficiently sensitive for detection in plasma, highlighting the need for better assays. For most subjects in the cohorts analyzed, imaging data and/or CSF samples were not available and thus could not be included in the analysis. Despite these limitations, we discovered and replicated evidence that neuroinflammation and complement dysregulation are pathological drivers in AD and thus potential therapeutic targets. Several observational studies have reported that long-term use of nonsteroidal anti-inflammatory drugs is associated with reduced risk of dementia [47], [48]; however, randomized controlled trials and systematic reviews found little or no benefit of nonsteroidal anti-inflammatory drugs [49], [50]. Perhaps, interventions in these latter studies were commenced too late to confer benefit. Inflammatory biomarkers to stratify and select patients for targeted early intervention might benefit future trials of anti-inflammatory interventions. Targeting complement dysregulation is, as yet, untested in AD. Although current anticomplement drugs are tailored for ultrarare diseases, numerous new drugs are progressing to the clinic, including for therapy of common inflammatory diseases, for example, age-related macular degeneration [51]. Anticomplement drugs designed to access brain and targeted to preclinical or early MCI patients identified and selected using markers of complement dysregulation may offer a new pathway to prevention of AD [52].

**Research in Context**

1. Systematic review: The authors reviewed the current literature using traditional (e.g., Google Scholar; PubMed) sources to identify published studies utilizing inflammation-relevant plasma biomarkers, in particular complement markers, for diagnosis, staging, or risk prediction of Alzheimer's disease. They noted the dearth of replicated plasma biomarkers and small sample size in many published studies.
2. Interpretation: Our findings identify sets of inflammatory biomarkers in plasma that distinguish clinical subgroups (controls: mild cognitive impairment; Alzheimer's disease) in a large multicenter cohort; these replicate in an independent cohort. Markers predictive of progression were also identified in the latter cohort.
3. Future directions: The findings require further replication in additional and larger independent cohorts, before development as a clinically viable multiplexed test for diagnosis and patient stratification.
